# Diet in Acute Nephritis

**Published:** 1915-02

**Authors:** 


### Diet in Acute Nephritis.

**Period of oliguria or anuria** (few days only):

| Article. | Amt. in 24 hrs. | Total Cal. | Grams, 24 hrs. P. | F. | C. | Water (c.c.) |
|---|---|---|---|---|---|---|
| Milk .................. | 1 pt. | 350 | 20 | 20 | 20 | 435 |
| Lime wtr. (dil. milk 1-3) | 150 c.c. | ... | .. | .. | .. | 150 |
| Totals .................... | | 350 | 20 | 20 | 20 | 585 |

**As soon as Kidneys secrete a moderate amount of urine:**

| | | | | | | |
|---|---|---|---|---|---|---|
| Milk .................. | 1½ pts. | 525 | 30 | 30 | 30 | 650 |
| Cream (20%) ......... | 4 oz. | 240 | 4 | 24 | 4 | 100 |
| Oatmeal gruel ......... | ½ pt. | 50 | 2 | 1 | 8 | 250 |
| Lime water ........... | 300 c.c. | ... | .. | .. | .. | 300 |
| Totals .................... | | 815 | 36 | 55 | 42 | 1300 |

**Diuresis established:**

| | | | | | | |
|---|---|---|---|---|---|---|
| Milk .................. | 1 qt. | 700 | 40 | 40 | 40 | 870 |
| Cream ................ | 6 oz. | 360 | 6 | 36 | 6 | 150 |
| Oatmeal gruel ......... | 1 pt. | 100 | 4 | 2 | 16 | 500 |
| Boiled rice ............. | 4 tbspn. | 240 | 4 | .. | 60 | ... |
| Cr. of Tartar water .... | 1 pt. | ... | .. | .. | .. | 500 |
| Totals .................... | | 1400 | 54 | 78 | 122 | 2020 |

**Convalescence established:**

| | | | | | | |
|---|---|---|---|---|---|---|
| Milk | 1 qt. | 700 | 40 | 40 | 40 | 870 |
| Cream | 6 oz. | 360 | 6 | 36 | 6 | 150 |
| Butter | 1 oz. | 240 | .. | 27 | .. | ... |
| Boiled rice | 4 tbspn. | 240 | 4 | .. | 60 | ... |
| Cooked cereal | 6 tbspn. | 200 | 6 | 3 | 36 | ... |
| Crackers | 2 oz. | 240 | 6 | 6 | 40 | ... |
| Sugar | 6 tspn. | 150 | .. | .. | 36 | ... |
| Water or Vichy | 1 pt. | ... | .. | .. | .. | 500 |
| Cr. of Tartar water | 1 qt. | ... | .. | .. | .. | 1000 |
| Totals | | 2130 | 62 | 112 | 218 | 2520 |

**Full Convalescence:**

| | | | | | | |
|---|---|---|---|---|---|---|
| Milk | 1 qt. | 700 | 40 | 40 | 40 | 870 |
| Eggs | 2 eggs | 160 | 16 | 11 | .. | ... |
| Cream | ½ pt. | 500 | 9 | 50 | 9 | 200 |
| Butter | 4 pats. | 320 | .. | 30 | .. | ..4 |
| Cooked cereal | 4 tbspn. | 135 | 4 | 2 | 24 | ... |
| Bread | 6 slices | 480 | 18 | 3 | 90 | ... |
| Sugar | 8 tspn. | 200 | .. | .. | 30 | ... |
| Baked apple | 200 gms. | 120 | .. | .. | 30 | ... |
| Water or Vichy | 1 qt. | ... | .. | .. | .. | 1000 |
| Cr. of Tartar water | 1 pt. | ... | .. | .. | .. | 500 |
| Totals | | 2615 | 87 | 142 | 243 | 2570 |

